# Curcumin inhibited HGF-induced EMT and angiogenesis through regulating c-Met dependent PI3K/Akt/mTOR signaling pathways in lung cancer

**DOI:** 10.1038/mto.2016.18

**Published:** 2016-08-03

**Authors:** Demin Jiao, Jian Wang, Wei Lu, Xiali Tang, Jun Chen, Hao Mou, Qing-yong Chen

**Affiliations:** 1 Department of Respiratory Disease, The 117th hospital of PLA, Zhejiang, P.R. China; 2 Department of Oncology, The 117th hospital of PLA, Zhejiang, P.R. China

## Abstract

The epithelial-mesenchymal transition (EMT) and angiogenesis have emerged as two pivotal events in cancer progression. Curcumin has been extensively studied in preclinical models and clinical trials of cancer prevention due to its favorable toxicity profile. However, the possible involvement of curcumin in the EMT and angiogenesis in lung cancer remains unclear. This study found that curcumin inhibited hepatocyte growth factor (HGF)-induced migration and EMT-related morphological changes in A549 and PC-9 cells. Moreover, pretreatment with curcumin blocked HGF-induced c-Met phosphorylation and downstream activation of Akt, mTOR, and S6. These effects mimicked that of c-Met inhibitor SU11274 or PI3 kinase inhibitor LY294002 or mTOR inhibitor rapamycin treatment. c-Met gene overexpression analysis further demonstrated that curcumin suppressed lung cancer cell EMT by inhibiting c-Met/Akt/mTOR signaling pathways. In human umbilical vein endothelial cells (HUVECs), we found that curcumin also significantly inhibited PI3K/Akt/mTOR signaling and induced apoptosis and reduced migration and tube formation of HGF-treated HUVEC. Finally, in the experimental mouse model, we showed that curcumin inhibited HGF-stimulated tumor growth and induced an increase in E-cadherin expression and a decrease in vimentin, CD34, and vascular endothelial growth factor (VEGF) expression. Collectively, these findings indicated that curcumin could inhibit HGF-promoted EMT and angiogenesis by targeting c-Met and blocking PI3K/Akt/mTOR pathways.

## Introduction

Lung cancer is the leading cause of cancer-related mortality worldwide. The prognosis of lung cancer is poor because lung cancer can be symptomless in the early stage. Therefore, searching new therapeutic agents and exploring novel intervention targets might provide more clinical benefits in lung cancer therapy.

Increasing evidence has shown that epithelial-mesenchymal transition (EMT) is associated with cancer development and metastasis.^1^ Cancer cells with EMT phenotype change often involve in epithelial characteristics loss and mesenchymal properties acquisition, exhibiting enhanced motility, and invasive abilities.^2^ A typical characteristic of EMT process is the mesenchymal markers, such as vimentin increased, while epithelial markers decreased like E-cadherin, which induces disruption of cell-to-cell junctions. EMT can be induced by various growth factors. Among them, hepatocyte growth factor (HGF) (also known as scattering factor) activates the c-Met signaling pathway, thereby increasing the invasive and metastatic potentials of the cells and allowing the survival of cancer cells in the bloodstream in the absence of anchorage.^3,4^ The clinical importance of HGF and its receptor c-Met has been further demonstrated in recent studies, showing that the levels of c-Met in mammary cancer tissues and levels of circulating HGF in patients with mammary cancer are associated with a lower survival and development of distant metastasis.^5,6^ In addition, HGF is well known as a potent angiogenic cytokine, and c-Met signal activation can modify the microenvironment to facilitate cancer progression.^3,4^ Moreover, HGF plays an important role in angiogenesis by cooperating with vascular endothelial growth factor, which is thought to be an important therapeutic target in lung cancer.^7^ Previously reported that HGF stimulated vascular endothelial growth factor production by EGFR-mutant lung cancer cells, thereby facilitating angiogenesis and tumor growth in xenograft models.^8^

Recently, the HGF/c-Met signaling pathways responsible for invasive growth have been mostly elucidated. The downstream signaling components include the PI3K/Akt, Ras/MAPK and the JAK/STAT pathway. Activation of these pathways is associated with increased scattering/motility, invasion, proliferation, survival, and angiogenesis.^9,10^ The interaction of PI3K with activated c-Met may enhance PI3K activity that has been implicated in the form of EMT and angiogenesis required for cell motility. Therefore, the HGF/c-Met signaling pathway is regarded as a promising therapeutic target, and many molecular targeted drugs are under clinical development.^11^

Curcumin (diferuloylmethane), the active component of the spice turmeric (Curcuma longa), has chemo-preventive and therapeutic properties against many tumors both *in vitro* and *in vivo*.^12,13^ Several studies have shown that curcumin induces apoptosis more potently in cancer cells than in normal cells and attributed its effect to the inhibition of angiogenesis, nitric oxide synthase, receptor tyrosine kinase and protein kinase C activities, and regulation of certain gene transcriptional factors, such as c-Jun/AP-1, JNK, nuclear factor κB, and P53.^14–16^ Previous studies from our lab showed that curcumin inhibited cell proliferation and induced cell apoptosis in lung cancer through the modulation of lysosomal pathway and reactive oxygen species-dependent mitochondrial signaling pathway.^12,17^ Furthermore, some studies showed that curcumin significantly inhibited the invasion and metastasis of tumors.^18–22^ Recently, some studies found that curcumin suppressed doxorubicin or LPS induced EMT in breast cancer and elucidated the role of TGF-β/PI3K/Akt and NF-κB-Snail signaling pathways in this process.^23,24^ Additionally, curcumin was shown to inhibit vascular endothelial cell proliferation *in vitro* and capillary tube formation *in vivo*.^25,26^ These studies demonstrated that the potent chemopreventive activity of curcumin may be partly derived from the direct inhibition of angiogenesis and EMT.

The aim of this study was to investigate the potential of curcumin to inhibit HGF-triggered EMT and angiogenesis in lung cancer cells *in vitro* and *in vivo*. Our data suggest that curcumin inhibits HGF-induced EMT and angiogenesis and this effect is accompanied by the inhibition of the c-Met/PI3K/Akt/mTOR signaling pathway.

## Results

### Curcumin uptake by cultured A549 and PC-9 cells

To examine curcumin uptake by A549 and PC-9 cells, the intrinsic fluorescence of curcumin was monitored by flow cytometry and fluorescence spectrophotometer. We first incubated A549 cells and PC-9 cells with curcumin (20 μmol/l) for 5 minutes, 15 minutes, 45 minutes, 1 hour, 1.5 hours, 2 hours, 4 hours, 6 hours, and 8 hours. Then, fluorescence spectrophotometer detection showed a rapid uptake of curcumin in these cells. Intracellular curcumin levels were maximal within 45 minutes (Supplementary Figure S1a,b). Furthermore, flow cytometry measurement of curcumin fluorescence in A549 cells treated with different amounts of curcumin (0–80 μmol/l external concentration) for 45 minutes showed that curcumin induced a concentration-dependent shift in cellular fluorescence. There was a significant linear relationship between curcumin concentration and cellular fluorescence (Supplementary Figure S1c). In addition, curcumin accumulation in cultured A549 cells and PC-9 cells was also detected by fluorescence spectrophotometer following incubation with increasing extracellular curcumin concentrations. The results showed that mean cellular curcumin fluorescence intensity was significantly increased at all examined. For external curcumin in A549 cells at 10, 20, and 30 μmol/l, the intracellular curcumin is of 0.47, 0.56, and 0.73 μmol/l, respectively (Supplementary Figure S1d). The same experiment was also performed in PC-9 cells. The results were showed in Supplementary Figure S1e.

### Curcumin inhibited HGF-induced lung cancer cell growth

**Figure 1:**
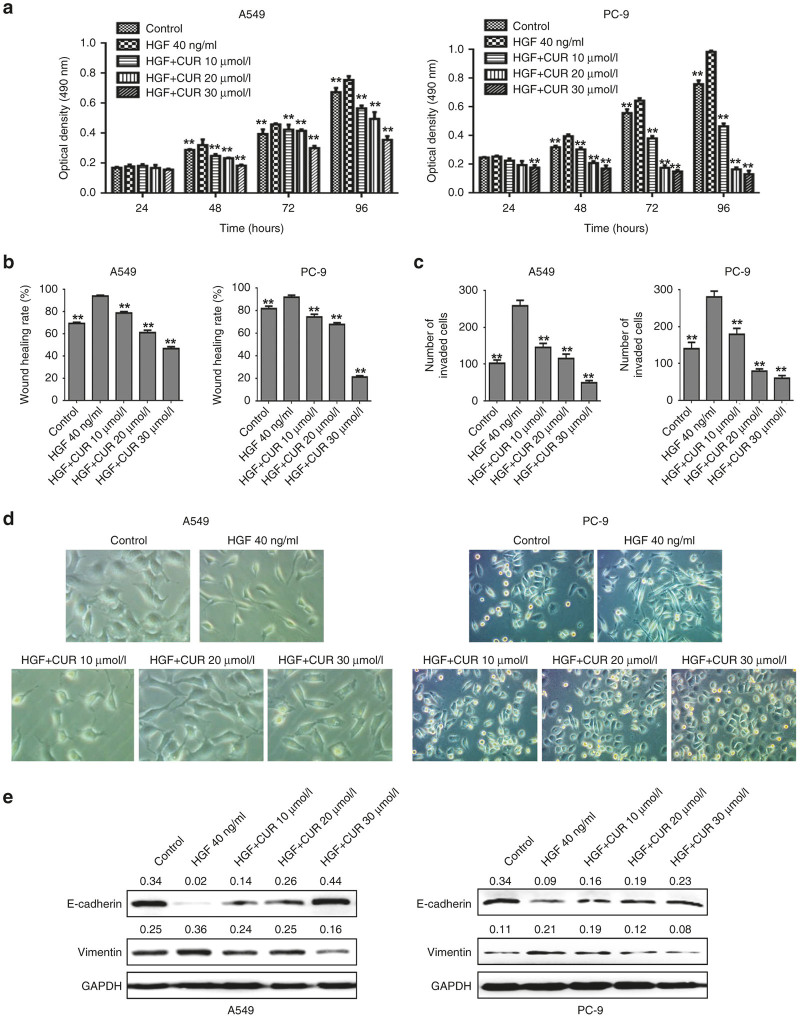
The effects of curcumin on hepatocyte growth factor (HGF)-induced cell proliferation, migration, invasion and epithelial-mesenchymal transition in lung cancer A549 and PC-9 cells. (**a**) A549 cells and PC-9 cells were starved for 12 hours and then stimulated by 40 ng/ml of HGF in the presence of 2% of fetal bovine serum for 24, 48, 72, and 96 hours. Cell proliferation was detected at indicted times. When curcumin was used, it was added 4 hours before HGF stimulation. **P < 0.01 compared with HGF group. (**b**) A549 cells and PC-9 cells were starved for 12 hours then both the cells were stimulated with 40 ng/ml of HGF in the presence of 2% of fetal bovine serum. Cell migration capability of A549 cells and PC-9 cells were determined by wound healing assay. When curcumin was used, it was added 4 hours before HGF stimulation. Data are means of three separated experiments ± SD, *P < 0.05, **P < 0.01 compared with HGF group. (**c**) The cells were added to the upper chamber in 2% fetal bovine serum (FBS) media and invaded toward 2% FBS and 40 ng/ml HGF containing growth media in the lower chamber. Invasion capability of A549 cells and PC-9 cells were determined by transwell assay. When curcumin was used, it was added to the upper chamber. Data are means of three separated experiments ± SD, *P < 0.05, **P < 0.01 compared with HGF group. (**d,e**) A549 cells and PC-9 cells were starved for 12 hours, then treated with 40 ng/ml of HGF (with 0.5% FBS) for 48 hours. The cells morphology (**d**) was observed by contrast microscopy (original magnification, ×200). The expression of E-cadherin and vimentin (**e**) were detected by western blotting analysis. When curcumin was used, curcumin was added 4 hours before HGF stimulation. Quantitative results are also illustrated. The data presents the average of three independent experiments; CUR, curcumin.

The activation of c-Met with HGF is known to play an important role in cell proliferation in many kinds of cancer cells.^3,4^ Therefore, we first investigated the role of HGF in the proliferation of lung cancer cells. A549 and PC-9 cells were treated with increasing concentrations of HGF (0, 10, 20, and 40 ng/ml) for 24, 48, 72, and 96 hours and MTT assays were performed. The results showed that HGF caused an increase in A549 and PC-9 cells proliferation and the maximum effect was identified at 40 ng/ml of HGF both in A549 and PC-9 cell lines (data not shown). Then the antiproliferative effects of curcumin on HGF-mediated growth of A549 and PC-9 lung cancer cells were detected. As shown in Figure 1a, curcumin treatment caused a significantly dose and time-dependent suppression effect on HGF-induced A549 and PC-9 cell proliferation.

### Curcumin inhibited HGF-induced lung cancer cell migration and invasion

Accumulating evidence has revealed that HGF contributes to increased metastatic progression in various context-dependent ways, including increased motility and invasiveness.^5,6^ To determine whether curcumin could prevent HGF-induced motility and invasiveness of A549 and PC-9 cells, wound healing assay and transwell assay were performed. As shown in Figure 1b. HGF-treated cells showed enhanced cell migration activity compared with that of control cells. However, migration activity of HGF-treated cells was significantly prevented by curcumin in a dose-dependent manner. Similarly, HGF promoted cell invasion ability in A549 and PC-9 cells compared with that of the control cells. In contrast, cotreated with HGF and curcumin impaired invasion of these cells (Figure 1c). These findings indicated that curcumin inhibited HGF-induced migration and invasion.

### Curcumin inhibited HGF-induced lung cancer cell EMT

Previous studies suggest that HGF as an independent factor may trigger the EMT process.^27^ To verify this phenomenon and determine whether curcumin could inhibit HGF-induced EMT, we used optical microscopy to investigate morphology changes of A549 and PC-9 cells exposed to HGF in the presence or absence of curcumin. As shown in Figure 1d, both cell types underwent typical EMT morphological changes in response to HGF, and acquired a spindle-shaped and fibroblast-like phenotype. There was a loss of cell-to-cell contact, resulting in scattered clusters of cells. However, the mesenchymal phenotype was much less evident in cells cotreated with HGF and curcumin compared with the cells treated with HGF alone. These results indicate that curcumin inhibits HGF-induced cell morphological changes of EMT.

To further confirm the effects of curcumin on HGF-induced EMT, we sequentially analyzed the expression of two EMT markers, E-cadherin and vimentin by western blotting. The results showed that the expression of E-cadherin protein was significantly down-regulated in the HGF group compared to the control, whereas vimentin protein expression was substantially increased (Figure 1e). Importantly, curcumin reversed HGF-induced EMT markers changes, causing reinduction of E-cadherin and reinhibition of vimentin expression in a concentration dependent manner, indicating that curcumin has inhibitory effects on HGF-induced lung cancer cell EMT.

### Curcumin inhibits HGF-induced EMT via modulation of c-Met dependent PI3K/Akt/mTOR signaling pathways

**Figure 2:**
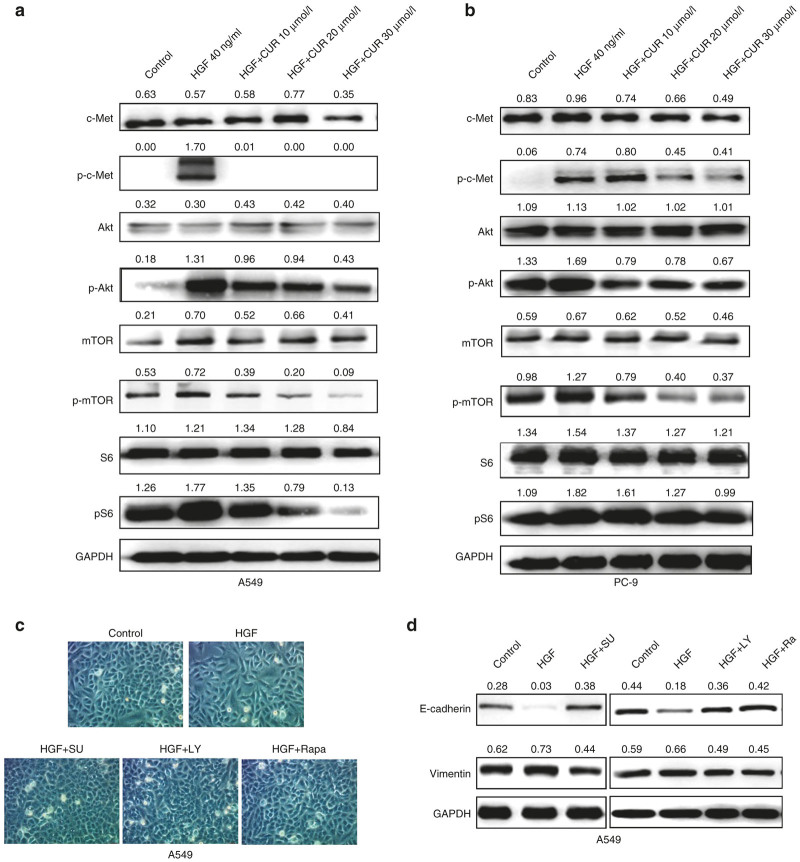
c-Met/PI3K/Akt/mTOR signaling pathway is involved in inhibition effect of curcumin on hepatocyte growth factor (HGF)-induced lung cancer cell epithelial-mesenchymal transition. A549 cells (**a**) and PC-9 cells (**b**) were starved for 12 hours and then stimulated with 40 ng/ml of HGF for 15 minutes with or without pretreated with different concentrations of curcumin (10–30 μmol/l) for 4 hours, protein expression of c-Met, p-c-Met, Akt, p-Akt, mTOR, p-mTOR, S6, and p-S6 were detected by western blotting analysis. Quantitative results are also illustrated. The data presents the average of three independent experiments. (**c,d**) A549 cells and PC-9 cells were starved for 12 hours, then treated with 40 ng/ml of HGF (with 0.5% fetal bovine serum) for 48 hours. MET inhibitors SU11274 (5 μmol/l), or PI3K inhibitor LY294002 (25 μmol/l), or mTOR inhibitor rapamycin (200 nmol/l) was used 4 hours before HGF stimulation. The cell morphology was observed by contrast microscopy (Original magnification, ×100) (**c**). The expression of E-cadherin, vimentin was detected by western blotting (**d**). Quantitative results are also illustrated. The data presents the average of three independent experiments. CUR, curcumin; SU: SU11274; LY: LY294002; Ra, Rapamycin.

Hepatocyte growth factor (HGF) can trigger cell migration through binding and activation of its only known receptor, c-Met, a receptor tyrosine kinase. The activated receptor serves as a docking site for various adaptor and signaling proteins, leading to the disassembly of adherents junctions, increasing cell motility, inducing EMT and angiogenesis.^9,10,28^ PI3K/Akt/mTOR signaling is one of the major pathways activated in cancer cells, including lung cancer cells. This pathway plays a variety of physiological roles, including regulation of EMT, cell growth, cell cycle, and cell survival.^9,10,29^ We have confirmed that HGF activated c-Met/PI3K/Akt/mTOR/S6 pathway in a concentration dependent manner (Supplementary Figure 2a,b). Therefore, we tested whether curcumin inhibited HGF-induced EMT via the inhibition of c-Met activation and PI3K/Akt/mTOR signaling. As shown in Figure 2a,b, Western blotting showed that 40 ng/ml HGF induced a rapid increase of c-Met, Akt, mTOR, and S6 phosphorylation levels in both A549 and PC-9 cells. Pretreatment of curcumin suppressed the HGF induced increase of c-Met, Akt, mTOR, and S6 phosphorylation in a concentration-dependent manner.

**Figure 3:**
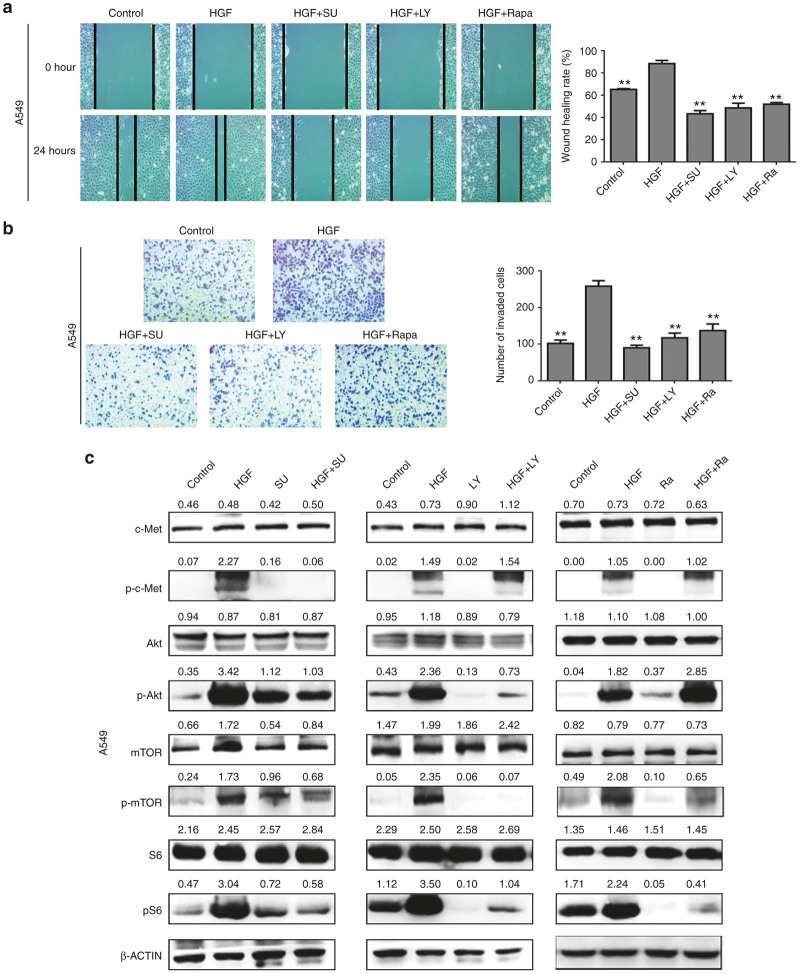
SU11274, LY294002, rapamycin inhibited hepatocyte growth factor (HGF)-induced lung cancer cell migration, invasion and c-Met/PI3K/Akt/mTOR signaling activation. (**a,b**) A549 cells were starved for 12 hours, then treated with 40 ng/ml of HGF (with 2% fetal bovine serum). MET inhibitors SU11274 (5 μmol/l), or PI3K inhibitor LY294002 (25 μmol/l), or mTOR inhibitor rapamycin (200 nmol/l) was used 1 hour before HGF stimulation. Cell migration capability was determined by wound healing assay (**a**). Invasion capability was determined by transwell assay (**b**). Data are means of three separated experiments ± SD, *P < 0.05, **P < 0.01 compared with HGF group. (**c**) A549 cells and PC-9 cells were starved for 12 hours, then treated with 40 ng/ml of HGF for 15 minutes. MET inhibitors SU11274 (5 μmol/l), or PI3K inhibitor LY294002 (25 μmol/l), or mTOR inhibitor rapamycin (200 nmol/l) was used 4 hours before HGF stimulation. Protein expression of c-Met, p-c-Met, Akt, p-Akt, mTOR, p- mTOR, S6, and p-S6 were detected by western blotting analysis. Quantitative results are also illustrated. The data presents the average of three independent experiments. SU, SU11274; LY: LY294002; Ra, Rapamycin.

To further detect the effects of c-Met/PI3K/Akt/mTOR signaling on HGF-induced EMT migration and invasion, A549 cells were treated with c-Met inhibitors SU11274, PI3K inhibitors LY294002, and mTOR inhibitor rapamycin respectively. As shown in Figure 2c,d, A549 cells stimulated by HGF are characterized by spindle-shaped morphology with few cell-cell contacts and a mesenchymal gene expression signature. In contrast, when these cells treated with three inhibitors, the mesenchymal appearance induced by HGF was rescued. In parallel, the expression of E-cadherin protein was significantly downregulated, vimentin protein expression was substantially increased in the HGF group compared to the control, whereas all the inhibitors reversed HGF-induced EMT, causing reinduction of E-cadherin and inhibition of vimentin expression (Figure 2d). Furthermore, we carried out wound-healing and transwell assays to determine the effects of three inhibitors on cell motility. As shown in Figure 3a,b, all of them could block HGF-enhanced migration and invasion. Importantly, similar results were also obtained in PC-9 cells (data not shown). These effects mimicked those of curcumin treatment.

In addition, we also detected the effect of c-Met inhibitors SU11274, PI3K inhibitors LY294002, and mTOR inhibitor rapamycin on the HGF-induced c-Met/PI3K/Akt/mTOR signaling in A549 cells. As shown in Figure 3c, HGF increased the c-Met, Akt, mTOR, and S6 phosphorylation, and which could be suppressed by SU11274. However, LY294002 only inhibited p-Akt and p-mTOR, and rapamycin only inhibited p-mTOR, indicating that c-Met/PI3K/Akt/mTOR signaling is a key pathway that involved in the roles of HGF in lung cancer, and might be also the main target of curcumin. Also in PC-9 cells, SU11274, LY294002, and rapamycin showed the similar results in suppressing the HGF-induced activation of c-Met/PI3K/Akt/mTOR signaling (data not shown).

**Figure 4:**
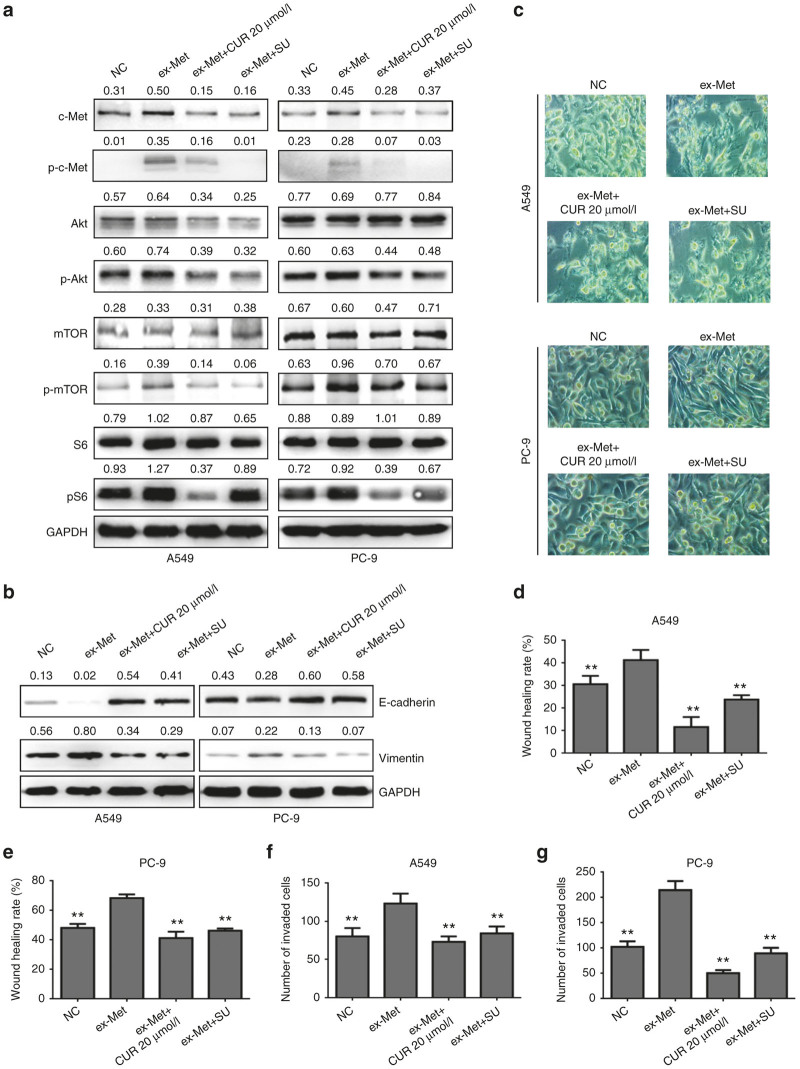
Curcumin could inhibit c-Met/PI3K/Akt/mTOR signaling activation, epithelial-mesenchymal transition, migration and invasion induced by c-MET overexpression in lung cancer A549 cells and PC-9 cells. A549 cells and PC-9 cells were transfected with c-Met overexpression plasmid (ex-MET) and then treated with 20 μmol/l of curcumin or 5 μmol/l of SU11274 for 24 hours, the expression of c-Met, p-c-Met, Akt, p-Akt, mTOR, p-mTOR, S6 p-S6, E-cadherin, and vimentin protein were detected by western blotting analysis (**a,b**). The cell morphology was observed by contrast microscopy (Original magnification, ×200) (**c**). Cell migration capability of A549 cells (**d**) and PC-9 cells (**e**) were determined by wound healing assay. Invasion capability of A549 cells (**f**) and PC-9 cells (**g**) were determined by transwell assay. Data are means of three separated experiments ± SD, **P < 0.01 compared with ex-Met group. NC, negative control; CUR, curcumin; SU, SU11274.

To further evaluate the relationship between curcumin inhibited EMT and the c-Met/Akt /mTOR pathway, we transiently transfected A549 and PC-9 cells with c-Met overexpression plasmid (ex-Met). The effect of c-Met overexpression on c-Met/PI3K/Akt/mTOR signaling was showed in Figure 4a. Compared with the control cells, the expression of c-Met was dramatically increased in ex-Met plasmid transfected cells. Meanwhile, ex-Met promoted Akt/mTOR/S6 activation. Combined treatment with ex-Met plasmid and curcumin significantly reduced Akt/mTOR/S6 phosphorylation level. Figure 4b–g also showed that cells overexpressing c-Met could promote EMT and migration, and the effects could be significantly inhibited by combined treatment with curcumin. Taken together, the effect of curcumin on HGF-induced EMT is through HGF/c-Met downstream signaling pathways.

### Curcumin induced apoptosis of HGF treated HUVECs and suppressed HGF-induced tubular structure formation and migration in HUVECs

Previous studies have reported that curcumin induced a significant apoptosis in human umbilical vein endothelial cells (HUVEC).^25,26^ To evaluate the death inducing capability of curcumin in HGF-treated HUVEC, we detected the cell viability and apoptosis under HGF stimulation with or without curcumin treatment. MTT assay showed that HGF significantly stimulated HUVEC proliferation and which could be efficiently inhibited by curcumin (Supplementary Figure S3a). Annexin V-EGFP/PI assay detected by flow cytometry showed that curcumin treatment for 24 hours increased HUVEC apoptosis and necrosis in a concentration dependent manner (Supplementary Figure S3b). The apoptosis rate was up to 21% when curcumin concentration at 30 μmol/l. Western blot assay also showed that the induction of apoptosis in response to curcumin, as did the elevated expression levels of Bax and cleaved PARP and the reduced levels of caspase-3, caspase-9, and Bcl-2 (Supplementary Figure S3c). These results indicated the pro-apoptosis roles of curcumin in HGF-treated HUVEC.

**Figure 5:**
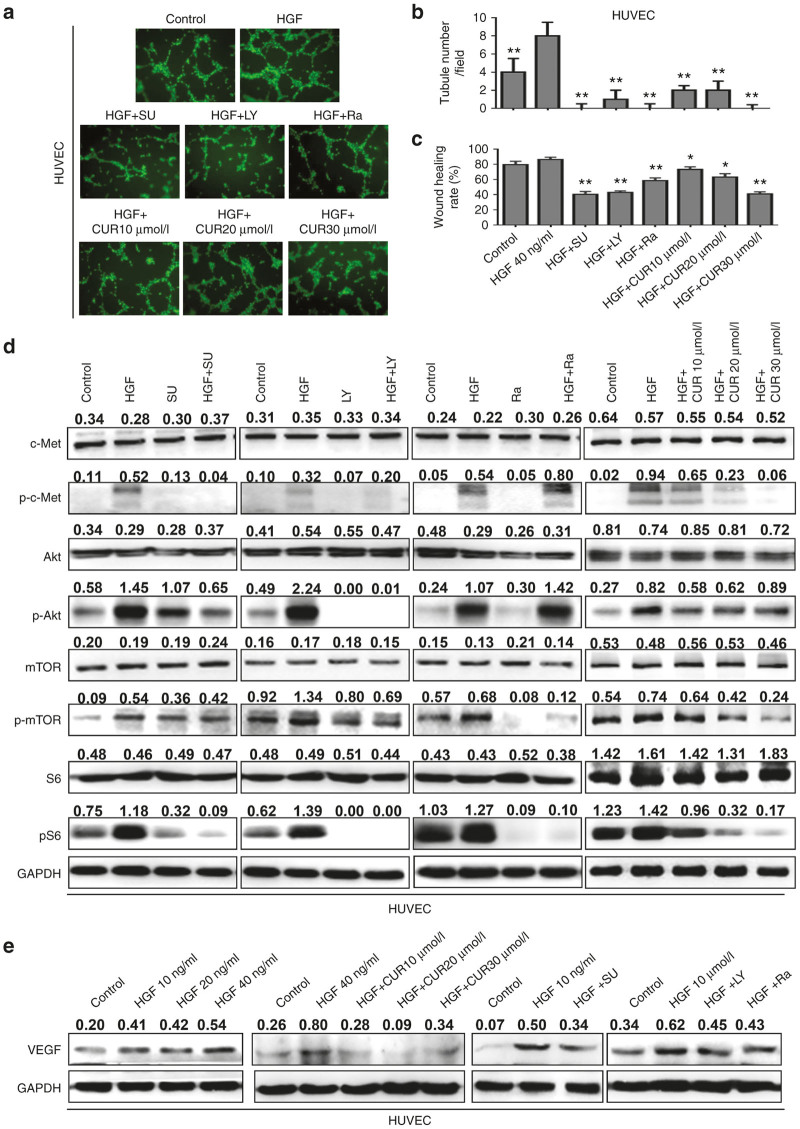
Curcumin suppresses the migration and tube formation of HUVEC cells by c-Met/Akt/mTOR pathway. (**a,b**) Representation (**a**) and quantification (**b**) of tube formation assay showing the angiogenic capability of HUVECs stimulated with hepatocyte growth factor (HGF) for 6 hours, or cotreated with different concentrations of curcumin (10–30 μmol/l) or different inhibitors (SU11274: 5 μmol/l, LY294002:25 μmol/l; rapamycin: 200 nmol/l). Original magniﬁcation: ×100. (**c**) Quantification of scratch migration assay showing the migration of HUVEC cells stimulated with HGF or combined with curcumin or different inhibitors. Data are means of three separated experiments ± SD, *P < 0.05, **P < 0.01 compared with HGF group. (**d**) HUVECs cells were starved for 12 hours, and stimulated with 40 ng/ml of HGF for 15 minutes. Different concentrations of curcumin or different inhibitors were used 4 hours before HGF stimulation. The cells were then harvested and lysed for the detection of p-c-Met, c-Met, p-Akt, Akt, p-mTOR, mTOR, S6, and p-S6. (**e**) HUVECs cells were treated with different concentrations of HGF for 24 hours, or pretreated with curcumin or different inhibitors for 4 hours. The vascular endothelial growth factor expression was examined by western blotting. Quantitative results are also illustrated. The data presents the average of three independent experiments. SU, SU11274; LY, LY294002; Ra, Rapamycin.

To further assess the antiangiogenic property of curcumin, we carried out capillary tube formation and migration assay. HUVECs were treated with 40 ng/ml HGF combined with or without various concentrations of curcumin (10–30 μmol/l) for 6 hours. Tube formation was visualized under a microscope. When the HUVECs were seeded on matrigel, robust tubular-like structures formed in the presence of HGF. However, pretreatment with curcumin significantly suppressed or terminated the HGF-induced formation of vessel-like structures as observed by the elongation and alignment of the cells at the indicated concentrations (Figure 5a,b). Cell migration is also critical for endothelial cells to form blood vessels in angiogenesis and is necessary for tumor growth and metastasis. Next, we conducted a wound migration assay to identify the effect of curcumin on HUVEC migration. When the endothelial cells were wounded and incubated in a medium with HGF in the presence of curcumin, HGF-induced cell migration was remarkably inhibited (Figure 5c). Therefore, curcumin showed an ability to block HGF-induced angiogenesis *in vitro*.

### Role of c-Met/PI3k/Akt/mTOR signaling in HGF-mediated HUVECs migration and angiogenesis

Hepatocyte growth factor (HGF)-mediated signaling promotes cell proliferation and migration in a variety of cell types. In the vasculature, c-Met activation via HGF plays an important role in regulating tumor angiogenesis.^30^ Recent studies indicate that PI3k/Akt pathway is involved in HGF-induced lung endothelial cells migration.^31^ To investigate the role of PI3k/Akt signaling in HGF-mediated angiogenesis, we used SU11274, LY294002, and rapamycin to inhibit c-Met/PI3k/Akt signaling respectively. The results were shown in Figure 5a,b. Three inhibitors significantly reduced HGF-induced tube formation and migration. Furthermore, western blotting showed that HGF induced c-Met phosphorylation, activation of Akt, mTOR, and S6 in HUVECs. The effects could be inhibited by curcumin in a dose-dependent manner. Similarly, SU11274, LY294002 could abolish HGF-induced Akt, mTOR, and S6 phosphorylation, and rapamycin could inhibit HGF-induced mTOR and S6 phosphorylation (Figure 5d). In addition, curcumin, SU11274, LY294002, and rapamycin could also inhibit HGF-induced vascular endothelial growth factor (VEGF) expression in HUVECs (Figure 5e). Thus, c-Met/PI3k/Akt signaling axis is important in HGF-induced tube formation and cell migration of HUVECs.

### Curcumin inhibited tumor growth, angiogenesis, and metastasis in tumor xenografts

**Figure 6:**
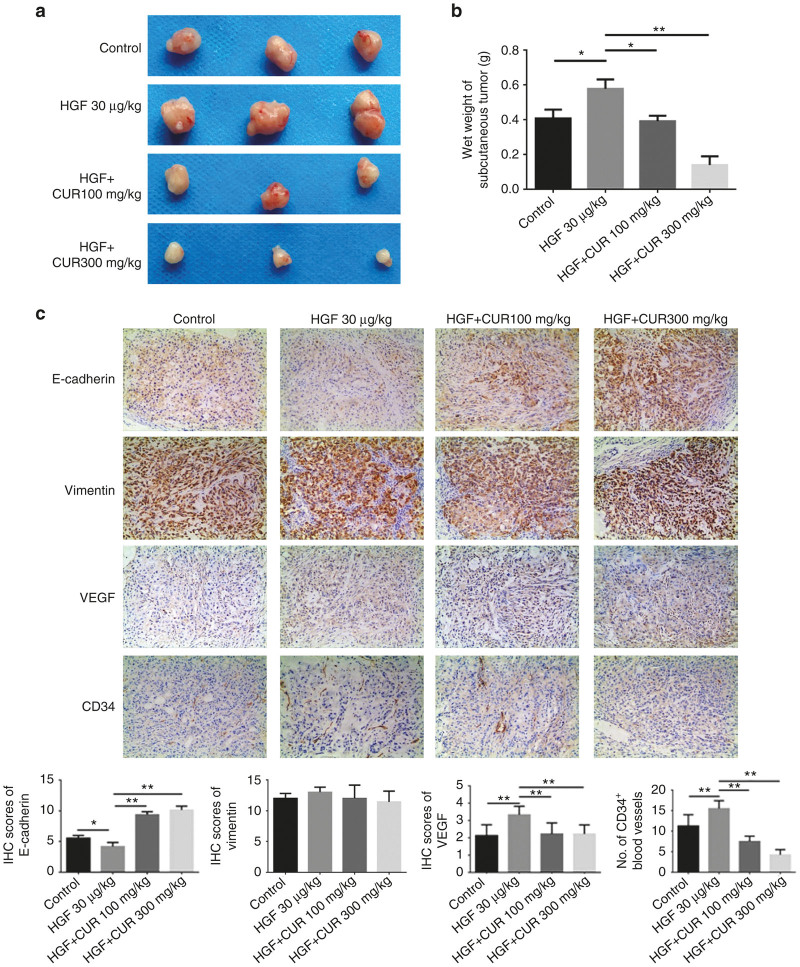
Curcumin effectively inhibited hepatocyte growth factor-induced tumor growth, epithelial-mesenchymal transition, vascular endothelial growth factor (VEGF) expression and angiogenesis in the xenograft tumor model of lung cancer. (**a**) Image of tumor size and (**b**) wet tumor weight at the time of dissection. (**c**) Immunohistochemistry (IHC) of the expression pattern of E-cadherin, vimentin, VEGF and CD34 using tumor tissues derived from xenograft tumor model. Representative images of each group are shown (Magnifications: 100×). Data are means ± SD; *P < 0.05. ** *P*<0.01.

To further determine whether curcumin has the same inhibitory effect in tumorigenesis, EMT and angiogenesis *in vivo*, a xenograft tumor model was used in the nude mice. As a result, HGF injection resulted in an increase of tumor size and weights. While combined treatment with HGF and curcumin showed a significantly inhibitory effect on tumors growth (Figure 6a,b). Tumor weights in HGF plus curcumin group were significantly lower compared with HGF group (0.39 ± 0.03 g in CUR100+HGF group, 0.13 ± 0.05 g in CUR300+HGF group versus 0.57 ± 0.05 g in HGF group). To analyze EMT and angiogenesis of tumors, tumor tissues were analyzed by immunohistochemical staining with anti-E-cadherin, anti-vimentin, anti-CD34, and anti-VEGF antibodies. The results showed that the expression of E-cadherin in the combined curcumin-treated groups was stronger than the HGF groups, while the expression of VEGF and CD34 in the curcumin-treated groups was lower than the HGF group (Figure 6c). Therefore, these data indicated the significant inhibitory roles of curcumin in HGF-stimulated lung cancer growth *in vivo*, the mechanisms would seem to inhibit EMT and VEGF-mediated angiogenesis.

## Discussion

Accumulated evidences suggested that HGF plays an important role as a regulator of the EMT process and accelerates the tumor-promoting activity in various cancer progressions involving the progression of metastatic lung cancer.^32,33^ Curcumin is a natural polyphenolic compound that has potent antiproliferative and antimetastasis effect on various cancers. In this study, we reported that curcumin not only inhibits HGF-induced EMT of lung cancer cells, but also suppresses angiogenesis of HUVECs, and the mechanism is related to regulation of c-Met-dependent PI3K/Akt/mTOR signaling pathways.

EMT comprises a complex series of reversible events that can lead to the loss of epithelial cell adhesion and the induction of a mesenchymal phenotype.^34^ HGF is a multifunctional cytokine that implicated in the EMT processes.^35,36^ Recent studies have implied that curcumin acts as crucial modulators for EMT.^37,38^ For example, curcumin suppresses doxorubicin-induced epithelial-mesenchymal transition via the inhibition of TGF-β and PI3K/Akt signaling pathways in triple-negative breast cancer cells.^23^ Curcumin also inhibits LPS-induced EMT through downregulation of NF-κB-Snail signaling in breast cancer cells.^24^ Other study showed that curcumin reversed the epithelial-mesenchymal transition of pancreatic cancer cells by inhibiting the Hedgehog signaling pathway.^39^ Furthermore, curcumin ameliorates epithelial to mesenchymal transition of podocytes *in vivo* and *in vitro* via regulating caveolin-1.^37^ However, the relation between curcumin, EMT and HGF/c-Met pathway remains unclear in lung cancer cells. Herein, we characterized and dissected a speciﬁc cascade in lung cancer cells. HGF stimulated the phosphorylation of c-Met followed by an increase in the levels of vimentin and decrease of E-cadherin. HGF-treated A549 and PC-9 cells have undergone EMT with enhanced invasiveness and motility. However, curcumin suppressed HGF-induced EMT. Curcumin treatment resulted in upregulation of E-cadherin, as well as downregulation of vimentin both in A549 cells and PC-9 cells. In parallel, curcumin suppressed the HGF-induced invasion and migration of A549 and PC-9. Therefore, we demonstrated that curcumin inhibits HGF-induced EMT in lung cancer cells.

Another major mechanism by which curcumin mediates anticancer effects against tumor appears to be the suppression of angiogenesis.^25,26^ The HGF/c-Met pathway is known to promote tumor angiogenesis thereby fostering cancer progression. Ponzo et al have reported that c-Met is expressed in endothelial cells and that HGF can stimulate endothelial cell growth, invasion and motility.^40^ Also, inhibition of c-Met activity impairs the survival, invasion, and tubulogenesis of HUVECs *in vitro* and reduces neovascularization and microvessel formation in tumor models.^41,42^ In accordance with this evidence, our results showed that curcumin at the low concentration could effectively inhibit endothelial cell migration and capillary structure formation in HGF stimulation models *in vitro*.

The cell surface receptor tyrosine kinase c-Met is upregulated in a variety of tumors, including NSCLC.^38^ c-Met is important in cell migration and invasion and correlates with prognostic parameters and poor survival in NSCLC.^43^ Increased c-Met signaling promotes cell migration and invasion through several pathways such as the focal adhesion kinase (FAK), phosphatidyl inositol 3-kinase (PI3K), and extracellular signal-regulated kinase (ERK) pathway.^44^ Furthermore, MET signaling dysregulation has been reported to be involved in tumor angiogenesis.^45^ PI3K/Akt/mTOR can also regulate many of the biological phenomena, including angiogenesis.^46^ In our study, we found that HGF stimulated phosphorylation of c-Met, Akt, and mTOR in human lung cancer cells and HUVECs. More importantly, curcumin suppresses HGF-induced phosphorylation of c-Met/Akt/mTOR in human lung cancer cells. Similarly, curcumin also suppresses HGF-induced phosphorylation of c-Met/Akt/mTOR in HUVECs. More importantly, c-Met inhibitor SU11274, PI3K inhibitor LY294002, and mTOR inhibitor rapamycin also inhibited HGF-induced phosphorylation of Akt/mTOR by downregulating c-Met and the effects was similar to that of curcumin, suggesting the important roles of c-Met/PI3K/Akt/mTOR pathway in the anticancer effect of curcumin.

To further confirm the effects of curcumin on EMT of lung cancer, we improved c-Met expression by transfecting A549 cells and PC-9 cells with c-Met overexpression plasmid and found that c-Met induced EMT in these cells. The cells with high c-Met expression displayed a spindle shape. E-cadherin expression was reduced, while vimentin expressions were increased. Importantly, curcumin could also suppress the EMT in c-Met overexpressed A549 and PC-9 cells. E-cadherin expression was increased and vimentin expressions were reduced after curcumin treatment. These results indicated that curcumin targeting c-Met and PI3K/Akt/mTOR pathway might be an important mechanism in reversing HGF-induced EMT in lung cancer cells.

In this study, we further extended our study to an *in vivo* xenograft model. Once time, we demonstrated that curcumin decreased the lung cancer cell proliferation. Indeed, immunohistochemical analysis further indicated that the expression of E-cadherin in the curcumin-treated groups was stronger or higher, while the expression of vimentin was lower than those in the control groups. Previous studies have reported that HGF promotes angiogenesis via the upregulation of VEGF.^30^ Loss of HGF signaling has been reported to reduce VEGF expression or its angiogenic potency.^47^ Consistent with these studies, VEGF and micro vessel density (MVD) were significantly decreased in the curcumin treatment group. These observations show that curcumin may block HGF-stimulated EMT and angiogenesis *in vivo*.

It has been reported that curcumin is able to target diverse mechanisms involved in the cell cycle, apoptosis, angiogenesis, invasion, and metastasis.^14–16,18,19,22^ However, how these mechanisms interconnected remain obscure. Recently, some researchers reported that curcumin induces crosstalk between autophagy and apoptosis mediated by calcium release from the endoplasmic reticulum, lysosomal destabilization, and mitochondrial events.^48,49^ These results indicated the complex interplay of antitumor mechanisms of curcumin. In our study, we found that curcumin could inhibit HGF-induced migration, EMT, and angiogenesis by the inhibition of the c-Met/PI3K/Akt/mTOR signaling pathway. Previous studies from our lab also showed that curcumin inhibited cell proliferation and induced cell apoptosis in lung cancer through the modulation of lysosomal pathway and reactive oxygen species-dependent mitochondrial signaling pathway.^12^ Therefore, curcumin induced apoptosis may also have a role for inhibition of HGF induced EMT and angiogenesis. In addition, our previous data have demonstrated that the concentrations of curcumin from 10 to 30 μmol/l have a significant pro-oxidant activity by increasing the generation of excessive reactive oxygen species.^12^ Furthermore, reactive oxygen species was reported to regulate EMT in cancer cells.^50^ Thus, these findings also revealed a possible link between pro-oxidant, proapoptosis, and anti-EMT activity of curcumin. Of course, the exact mechanisms need to be further studied.

In conclusion, our results suggest the evident effect of curcumin on suppressing HGF-induced EMT process and angiogenesis *in vitro and in vivo*. The mechanisms are included in inhibiting the activation of c-Met dependent PI3K/Akt/mTOR signaling and regulating the expression of the important markers E-cadherin, vimentin, and VEGF. These findings and concepts disclosed here provide an important basis for a further exploration toward understanding the action mechanisms of curcumin.

## Materials and Methods

### Cell culture

Human lung cancer cell line A549 was purchased from Chinese Academy of Sciences Cell Bank. HUVEC line was provided by Zhejiang Chinese Medical University. Human lung cancer cell line PC-9 was provided by Affiliated Hangzhou Hospital of Nanjing Medical University. All the cells were maintained in RPMI-1640 medium supplemented with 10% fetal bovine serum (Gibco, Grand Island, NY) in a humidified atmosphere containing 5% CO_2_ at 37 °C.

### Reagents and antibodies

Curcumin were purchased from Sigma Chemical Co (St. Louis, MO). HGF was purchased from Peprotech (Shanghai, China). c-Met inhibitor (SU11274), PI3 kinase inhibitor (LY294002) and rapamycin were purchased from Selleck Chemicals (Houston, TX). Primary antibodies phospho-c-Met (Y1234/35), phospho-Akt (S473), phospho-mTOR (S2448), c-Met, Akt, mTOR, S6, phospho-S6, E-cadherin, Vimentin, poly adenosinediphosphate-ribose polymerase (PARP), Bcl-2, Caspase-3, Caspase-9, Bax, β-ACTIN and glyceraldehyde-3-phosphate dehydrogenase (GAPDH) were purchased from Cell Signaling Technology (Beverley, MA). Secondary antibodies, Horse Radish Peroxidase (HRP) conjugated goat anti-mouse IgG and goat anti-rabbit IgG, were obtained from Jackson (West Grove, PA).

### Cell-uptake study

Flow cytometry (BD Accuri C6 BD Biosciences, New Jersey) and fluorescence spectrophotometer (Synergy Him & Take 3, BioTek, Vermont) were used to detect curcumin that cell uptake. For flow cytometry detection, A549 cells were plated at 1 × 10^6^ cells per well in six-well plates and allowed to attach overnight. After cells were incubated with curcumin (0–80 μmol/l) for 45 minutes, the cells were trypsinized and washed with phosphate-buffered saline (PBS), Internal curcumin fluorescence in different treatment groups were determined using flow cytometry. For fluorescence spectrophotometer detection, 1 × 10^6^ cells were plated in 24-well plates and allowed to attach overnight. After cells were incubated with curcumin (0–80 μmol/l) for 5 minutes, 15 minutes, 45 minutes, 1 hour, 1.5 hours, 2 hours, 4 hours, 6 hours, 8 hours, cells were spinned down and washed twice with PBS. For the estimation of cellular uptake, the pellet was dried and suspended in to 1 ml of methanol and sonicated till curcumin is completely extracted into the methanol fraction. The lysate was centrifuged at 10,000 rpm for 5 minutes, absorption spectra of supernatant containing methanolic curcumin were recorded and from the absorbance at 428 nm, the total cellular uptake was estimated.^51^

### MTT assay

A549 and PC-9 cells were seeded in 96-well plates (5 × 10^3^ cells/well) overnight and then serum-starved for 6 hours followed by treatment with HGF (0–40 ng/ml) for 48 hours. Forty-eight hours after the addition of the HGF, cell viability was assessed by the 3-(4,5-dimethylthiazol-2-yl)-2,5-diphenyl-tetrazolium bromide (MTT Beyotime, Shanghai, China) assay. The absorbance at 490 nm (A490) of each well was read on a spectrophotometer. When curcumin was used, the cells were pretreated with indicated concentrations of curcumin for 4 hours before HGF stimulation.

### Plasmid and transfection

c-Met (Accession NO.: NM_000245) overexpression vector (pEZ/M98/ neo-c-Met, designated as ex-Met) and the control vector (pEZ/M98/neo-control, designated as ex-NC) were provided by genecopoeia Inc (Guangzhou, China). The overexpression vectors were transfected by using Lipofectamine 2000 (Invitrogen, San Diego, CA) reagent according to the manufacturer’s instructions.

### Migration and invasion assay

Wound healing experiment and transwell assays were used to determine the migration and invasion abilities of the cells, respectively. For wound healing experiment, cells were grown to confluence in six-well plates, wounded and photographed at 0- and 24-hour time points. Cell migration was evaluated by measuring the width of the wound at the identical position. For the transwell assay, the lower chambers of matrigel-coated transwell insert (24-well insert; pore size 8 μm, Corning, New York) were used. Cells (100,000) were added to the upper chamber in serum-free media and invaded towards 10% fetal bovine serum-containing growth media in the lower chamber. After 24 hours, cells that invaded through the membrane were fixed, stained with crystal violet, and counted with light microscopy. All experiments were carried out in triplicate.

### Flow cytometry analysis for cell apoptosis

HUVEC were starved for 12 hours and then stimulated by 40 ng/ml of HGF in the presence of 2% of fetal bovine serum for 24 hours. When curcumin was used, 10–40 μmol/l curcumin were added 4 hours before HGF stimulation. HUVEC were collected after treated with curcumin for 24 hours and washed twice with PBS, then stained with Annexin V-EGFP/PI kit (Keygene Biotech, Nanjing, China) according to the manufacture’s instruction and analyzed by flow cytometry, 10,000 cells were counted.

### Western blot analysis

Cells were washed in PBS and lysed in RIPA lysis buffer supplemented with protease inhibitor cocktail (Roche, Mannheim, Germany). Total protein was quantified by BCA Protein Assay Kit (Beyotime, Nanjing, China), and an equal amount of whole cell lysates was resolved by sodium dodecyl sulfate-polyacrylamide gel electrophoresis and transferred to a polyvinylidene dfluoride membrane (Millipore, Darmstadt, Germany). The blots were blocked in bovine serum albumin (BSA) (5% w/v in PBS + 0.1% Tween 20) for 1 hour at room temperature and immunostained with antibodies at 4 °C overnight. Immunoreactive bands were visualized by enhanced chemiluminescence (Millipore Darmstadt, Germany) according to the manufacturer’s instructions. Data were normalized to GAPDH or β-ACTIN.

### Capillary tube formation analyses

HUVECs were cultured at 37 °C in a 96-well plate coated with Matrigel (BD Pharmingen, San Diego, CA). After being treated with indicated concentrations of HGF or inhibitors for indicated time, the formation of capillary-like structures was AO (acridine orange, Shanghai, China) stained and captured under a light microscope. The number of the formed tubes, which represent the degree of angiogenesis *in vitro*, was scanned and quantitated in ﬁve low power ﬁelds (100× magniﬁcation).

### Animal studies

All experimental procedures used in this study had been approved by the ethics committee in the 117th Hospital of PLA. Male nude mice (BALB/c, 4-6wk) were purchased from Shanghai Laboratory Animal Center (Shanghai, China). For preparation of subcutaneous xenograft model, A549 cells (1 × 10^7^) in phosphate-buffered saline/100 μl were injected subcutaneously into the right flank of each mouse. Fifteen days after cell inoculation, total of 12 mice were divided randomly into four groups. 100 or 300 mg/kg curcumin was administrated every day. While intratumoral injection of PBS or 30 µg/kg HGF was performed every 3 days. After 30 days of treatment, all mice were sacrificed. Transplanted tumors were excised, and the wet weight was recorded. Protein expression in tumors was detected by immunohistochemistry method.

### Immunohistochemistry

Tissue slides were incubated for 2 hours at 56 °C and deparaffinized. Antigen retrieval was obtained by microwave treatment in citrate buffer for 15 minutes to retrieve antigenicity. After peroxidase activity was blocked with 3% H_2_O_2_/methanol for 10 minutes, sections were incubated with normal goat serum for 20 minutes to block nonspecific antibody binding sites. Sections were incubated with the primary antibodies for 1 hour at 25 °C followed by incubations with biotinylated anti-rabbit/mouse IgG and peroxidase-labeled streptavidin for 10 minutes each. The percentage of the cells with cytoplasmic labeling was recorded from two areas of each specimen, and the labeling intensity was estimated as 1+, 2+, or 3+. The immunohistochemistry results were categorized into two groups: the samples without any labeling, 1+ labeling in <25% cells, and 2+ labeling in <5% cells were considered negative; all the remaining samples were defined as positive.

### Statistical analysis

All statistical analyses were performed using SPSS 13.0 (SPSS Inc, Chicago, IL). Numerical data were presented as mean ± SD. The statistical difference of data between groups was analyzed by one-way analysis of variance and Student’s *t*-test. Differences were considered significant when *P* < 0.05.

## Notes

The first three authors contributed equally to this work.
