# Supplementary material for: Curcumin inhibited HGF-induced EMT and angiogenesis through regulating c-Met dependent PI3K/Akt/mTOR signaling pathways in lung cancer
Source: Mol Ther Oncolytics. 2016 Aug 3;3:16018–. doi: 10.1038/mto.2016.18 (PMC4972091; doi:10.1038/mto.2016.18)
Supplement: Supplementary Figures [file mto201618-s1.zip › mto-00082-s01.docx]

**Supplementary figure. 1 Cellular uptake and intracellular concentration of curcumin.** (a-b) A 8 h time course showing the rapid uptake of curcumin in A549 cells (a) and PC-9 cells (b). Intracellular curcumin levels were maximal within 45 min. (c)Flow cytometry measurement of curcumin fluorescence in A549 cells treated with different amounts of curcumin (0–80μM external concentration). The plotted mean values were recorded after 45 min of equilibration. (d-e) Intracellular curcumin concentration in μM, as measured by methanolic extraction, of A549 cells (d) and PC-9 cells (e) treated with various concentrations of curcumin plotted against the external concentration of curcumin (0–80μM; n=6 independent experiments). Cells without treatment were used as negative controls (to determine cellular autofluorescence at the wavelength used, i.e., 530±30nm).

**Supplementary figure. 2 HGF activated c-Met/PI3K/Akt/mTOR/S6 pathway in a concentration dependent manner both in A549 cells and PC-9 cells.** A549 cells (a) and PC-9 (b) cells were starved for 12h, then treated with different concentrations of HGF for 15 min. Protein expression of c-Met, p-c-Met, Akt, p-Akt, mTOR, p-mTOR, S6 and p-S6 were detected by western blotting analysis. Quantitative results are also illustrated. The data presents the average of three independent experiments.

**Supplementary figure. 3 Curcumin inhibited HGF-induced HUVEC proliferation and induced apoptosis of HUVEC.** (a) HUVEC were starved for 12h and then stimulated by 40 ng/ml of HGF in the presence of 2% of fetal bovine serum for 24h, 48h, 72h and 96h. Cell proliferation was detected at indicted times. When curcumin was used, 10-30μM were added 4 h before HGF stimulation. ** P<0.01 compared with HGF group. (b) HUVEC cells were collected and stained with annexin V-EGFP and PI after treated with HGF with or without curcumin for 24 hours, then determined by flow cytometry; (c) HUVEC cells were treated with HGF with or without curcumin for 24 hours, then cell lysates were collected and western blot was conducted for the indicated (apoptosis related) proteins; Quantitative results are also illustrated. The data presents the average of three independent experiments. CUR: curcumin;
