# Supplementary figures and images for: Curcumin inhibited HGF-induced EMT and angiogenesis through regulating c-Met dependent PI3K/Akt/mTOR signaling pathways in lung cancer
Source: Mol Ther Oncolytics. 2016 Aug 3;3:16018–. doi: 10.1038/mto.2016.18 (PMC4972091; doi:10.1038/mto.2016.18)

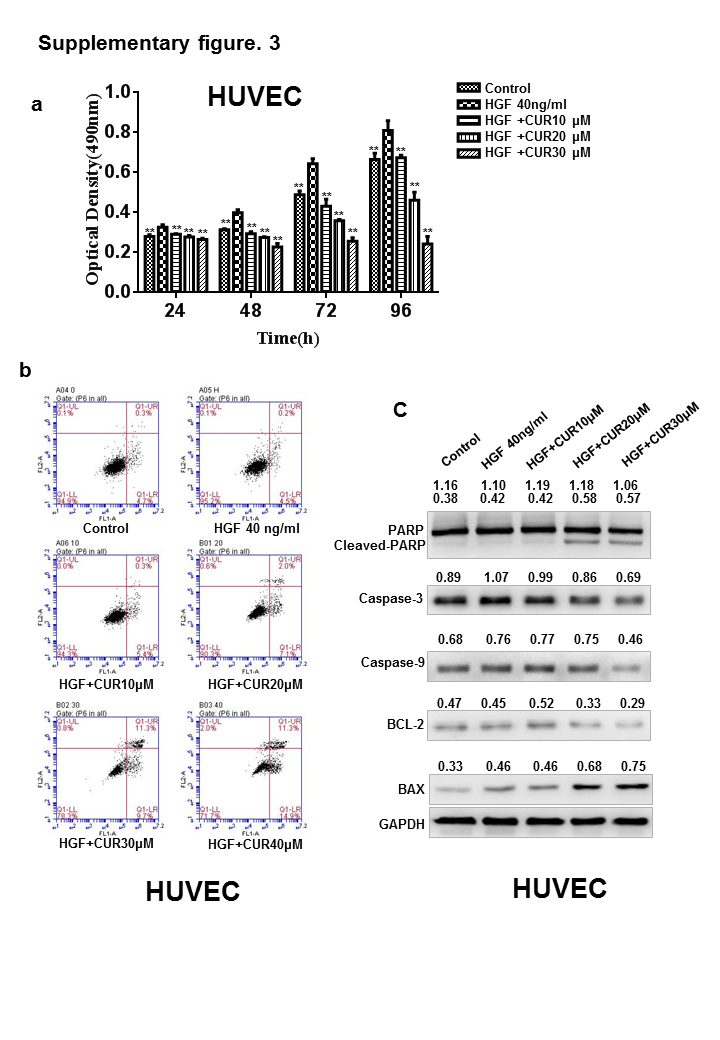

Supplement: Supplementary Figures [file mto201618-s1.zip › mto-00082-s04.tif]

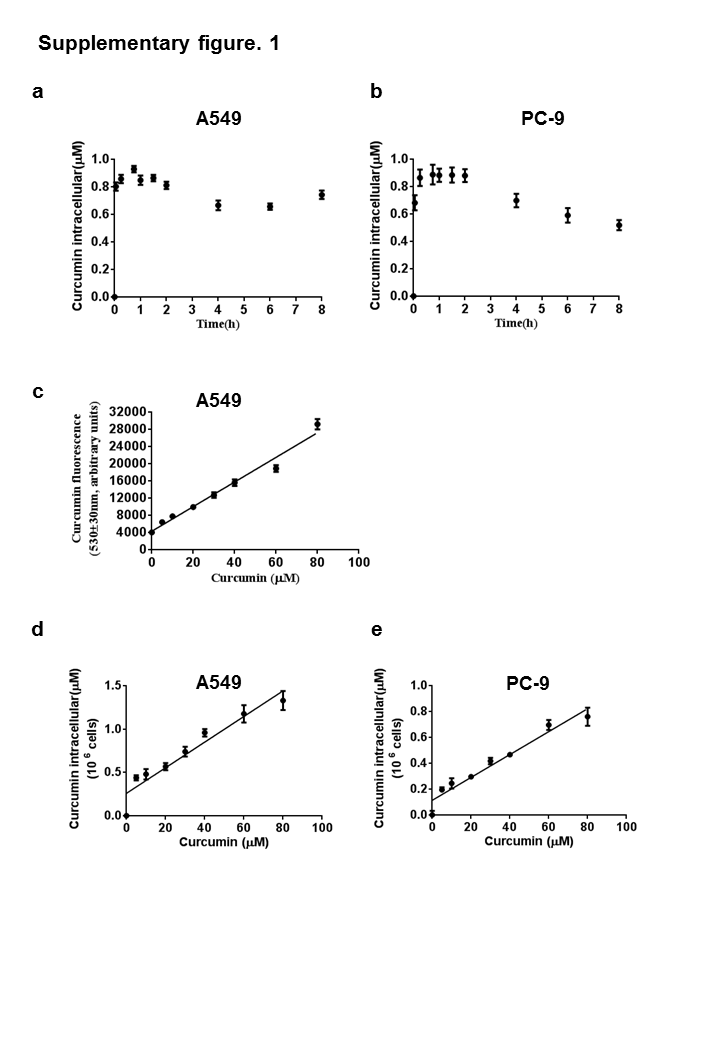

Supplement: Supplementary Figures [file mto201618-s1.zip › mto-00082-s02.tif]

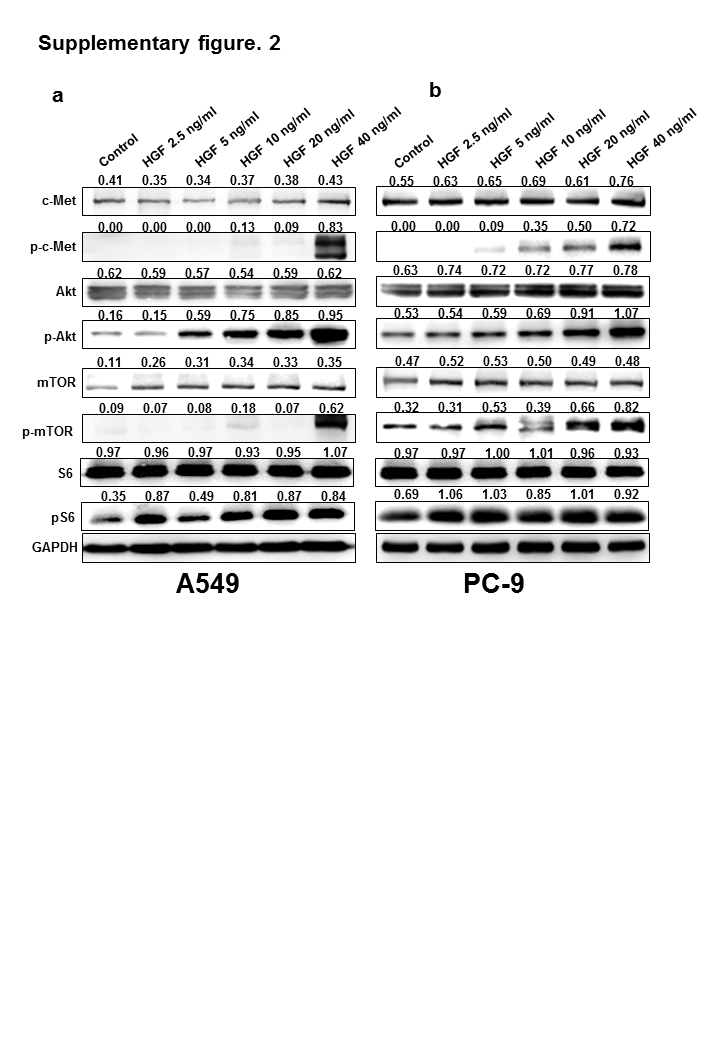

Supplement: Supplementary Figures [file mto201618-s1.zip › mto-00082-s03.tif]
